# Case Report: renal allograft infiltration by chronic lymphocytic leukemia successfully treated with zanubrutinib

**DOI:** 10.3389/fonc.2026.1765255

**Published:** 2026-02-02

**Authors:** Brian Abboud, Nehemias Guevara Rodriguez, Arslan Babar, Noemy Coreas, Katherine Robbins, Ranju Kunwor

**Affiliations:** 1 Department of Internal Medicine, SSM Health-Saint Louis University School of Medicine, Saint Louis, MO, United States; 2 Division of Hematology, Oncology and Bone Marrow Transplant, Department of Internal Medicine, SSM-Health Saint Louis University School of Medicine, St. Louis, MO, United States; 3 Department of Gynecologic Oncology, Salvadoran Social Security Institute (ISSS), University of El Salvador (UES), San Salvador, El Salvador; 4 Department of Pathology, SSM-Health Saint Louis University School of Medicine, Saint Louis, MO, United States

**Keywords:** bruton tyrosine kinase (BTK) inhibitor, CLL (chronic lymphocytic leukemia), hematology, immunosuppression, malignant hematologic disease, renal transplant, zanubrutinib, zanubrutinib acute kidney injury as initial manifestation of chronic lymphocytic leukemia/small lymphocytic lymphoma

## Abstract

**Background:**

Chronic lymphocytic leukemia (CLL) is the most common leukemia in adults. It is characterized by the accumulation of mature monoclonal B lymphocytes. While native kidney infiltration in CLL is relatively common and typically subclinical, involvement of a transplanted kidney is exceedingly rare and may have profound clinical implications.

**Case Summary:**

We present the case of a 65-year-old woman with end-stage renal disease secondary to hypertension and diabetes mellitus who underwent kidney transplantation in 2016. Several years later, she was diagnosed with Rai stage I CLL following imaging and histopathologic analysis of axillary lymphadenopathy. Despite an initially indolent course, she developed worsening renal function in 2025. A biopsy of the allograft revealed CLL infiltration consistent with her prior nodal disease. The patient was started on Zanubrutinib, a Bruton tyrosine kinase inhibitor (BTKi), with stabilization of renal function.

**Conclusion:**

This case highlights a rare and clinically significant presentation of CLL involving a renal allograft. As the therapeutic landscape evolves with the advent of BTK inhibitors, prompt recognition and treatment of extranodal CLL involvement may improve outcomes. This case represents only the second reported instance of successful BTKi use for CLL infiltration in a kidney transplant recipient.

## Introduction

Chronic lymphocytic leukemia (CLL) is the most common type of leukemia in adults, representing nearly 25% of all newly diagnosed leukemia cases in the United States (1). CLL is characterized by the progressive accumulation of small, mature-appearing monoclonal B lymphocytes that coexpress CD5, CD19, CD23, and surface immunoglobulin light chains (2). The clinical course of CLL is heterogeneous, ranging from indolent forms requiring no treatment for years to rapidly progressive disease involving multiple organs. Staging systems such as Rai and Binet are commonly used to stratify disease severity and predict prognosis (2).

Although bone marrow, lymph nodes, spleen, and liver are the classic sites of CLL involvement, renal manifestations—particularly infiltration of the kidneys—are more frequent than clinically appreciated. Barcos et al. reported that in the autopsy series of CLL patients, renal infiltration was present in 63% of CLL cases, second only to the liver, spleen, and lymph nodes (3). However, native kidney involvement is often subclinical and rarely leads to overt renal dysfunction. In contrast, when CLL infiltrates the kidneys symptomatically, patients may present with acute kidney injury (AKI) and membranoproliferative glomerulonephritis (MPGN) often presenting as nephrotic syndrome (4). Renal complications of CLL can arise from multiple mechanisms, including direct leukemic infiltration, immune complex-mediated injury, and tumor lysis syndrome. In a comprehensive case series by Strati et al., more than 70% of renal pathologies in CLL patients were attributable to the leukemia itself, including glomerulonephritis, interstitial nephritis, and direct leukemic infiltration (4). Notably, renal dysfunction in these cases was not always proportional to peripheral lymphocyte counts, highlighting the need for tissue diagnosis when clinical suspicion is high.

While native kidney involvement in CLL has been well described, reports of leukemic infiltration of transplanted kidneys are exceedingly rare. A four-patient case series by D’Ythurbide et al. investigated outcomes of CLL in renal transplant recipients. They highlighted frequent progression of disease and high rates of infectious complications, with one patient losing the graft within 14 months due to leukemic infiltration (5). These outcomes were attributed not only to leukemic burden but also to immunosuppression-induced vulnerability.

Notably, many of these previously reported cases occurred before the advent of Bruton tyrosine kinase inhibitors (BTKi), which have since transformed the treatment landscape of CLL. Stavart et al. recently described a biopsy-confirmed CLL infiltration in a kidney allograft treated successfully with ibrutinib, resulting in clinical and histological remission (6). Our report builds upon this experience and represents, to our knowledge, the second case of CLL infiltration in a renal allograft treated with a second-generation BTKi, zanubrutinib.

Given the increasing use of kidney transplantation and the aging population at risk for CLL, awareness of this rare but important clinical entity is crucial. Early recognition, histological confirmation, and timely initiation of targeted therapy may help preserve graft function and improve outcomes in this vulnerable population.

## Case presentation

We present the case of a 65 year old woman with end-stage renal disease (ESRD), attributed to longstanding hypertension and poorly controlled Type 2 Diabetes Mellitus. After one year on peritoneal dialysis, she underwent a living unrelated donor kidney transplant in the mid-2010s. Pre-transplant serum creatinine (sCr) ranged from 1.0 to 1.2 mg/dL, with immediate posttransplant sCr between 1.0 and 1.4 mg/dL, indicating prompt graft function. The donor-recipient human leukocyte antigen (HLA) mismatch was 2A/2B/1DR, reflecting two mismatches at the HLA-A locus, two at HLA-B, and one at HLA-DR. The patient was cytomegalovirus (CMV) seropositive and received a kidney from a CMV-seronegative donor; she was also Epstein-Barr virus (EBV) seropositive. Due to the degree of HLA mismatch (Panel Reactive Antibody 0%) and the CMV mismatch status, both were considered to increase immunologic risk. Induction immunosuppression with three doses of thymoglobulin 1.5mg/kg was administered to reduce the risk of early acute rejection. Her maintenance immunosuppression consists of tacrolimus 2mg daily, Mycophenolate mofetil 150 mg twice daily, and prednisone 5mg daily, which she continues to receive.

One-year post-transplantation, the patient’s follow-up was complicated by worsening renal function. A subsequent renal biopsy revealed acute cellular rejection (ACR) and CD4+ antibodymediated rejection (AMR). This was treated with thymoglobulin 6 mg/kg, methylprednisolone sodium succinate 500 mg for three doses, five sessions of therapeutic plasma exchange, intravenous immunoglobulin (IVIG) 1g/kg for two doses, and rituximab 375mg/m^2^. BK virus test at this time proved negative. Despite continued maintenance immunosuppression with prednisone 5mg daily and tacrolimus 5 mg twice daily (trough 4ng/ml), poor adherence to her medication regimen led to recurrent presentations with impaired renal function in early 2018 (sCr 1.7-2.1 mg/dL) and early 2019 (sCr 1.9 mg/dL). Renal biopsies performed during these admissions demonstrated ongoing antibodymediated rejection. Treatment per the patient’s nephrologist included a 6-week methylprednisolone sodium succinate taper, five sessions of therapeutic plasma exchange, two doses of IVIG, rituximab 375mg/m^2^, and four doses of Bortezomib 2.5mg. Following the resolution of her renal impairment, she resumed her maintenance immunosuppression.

In late 2023, during a routine nephrology clinic follow-up, the patient’s complete blood count (CBC) revealed leukocytosis with a white blood cell count of 15x10^(E9)/L with a predominance of lymphocytes. Given the predominance of lymphocytes, a peripheral smear was ordered and reviewed by pathology, demonstrating lymphocytosis and smudge cells, raising concern for a neoplastic process, and prompting referral to hematology/oncology. Concurrently, a routine mammogram showed bilateral axillary adenopathy. Further evaluation with a computed tomography (CT) scan of the chest revealed widespread adenopathy in the mediastinum, axillae, supraclavicular, and hilar stations, prompting a lymph node biopsy. The biopsy in early 2024 confirmed Chronic Lymphocytic Leukemia (CLL). Flow cytometric immunophenotypic analysis of the right axillary lymph node showed that the majority of cells (~84% of events) were monoclonal B-cells expressing CD5 (dim), CD19, CD20 (dim), CD23, and surface kappa light chains, consistent with CD5- positive mature B-cell lymphoma and highly suggestive of chronic lymphocytic leukemia (CLL). Neoplastic cells were negative for CD10. T-cells showed no immunophenotypic aberrancy, with a CD4:CD8 ratio of 2.3:1. Chromosome analysis of the axillary lymph node revealed trisomy 12 with a t (14,19) translocation and additional material of uncertain origin on the short arm of chromosome 2 and gain of an isochromosome for the long arm of chromosome 17. IgHv mutation analysis was negative. At this time, given the patient’s Rai stage I and a CLL-International Prognostic Index (CLL-IPI) calculated at 5 points (high-risk category, 63.3% 5-year survival), the decision was made for continuous observation without therapy, as per International Workshop on Chronic Lymphocytic Leukemia (iwCLL) guidelines, which indicate no survival benefit from intervention in early-stage disease. Further follow-up with positron emission tomography/computed tomography (PET/CT) scans in mid-2024 and early 2025 showed innumerable small lymph nodes above and below the diaphragm; however, none were greater than 10 cm or showed concerns for transformation, thus indicating no immediate need for treatment.

**Figure 1:**
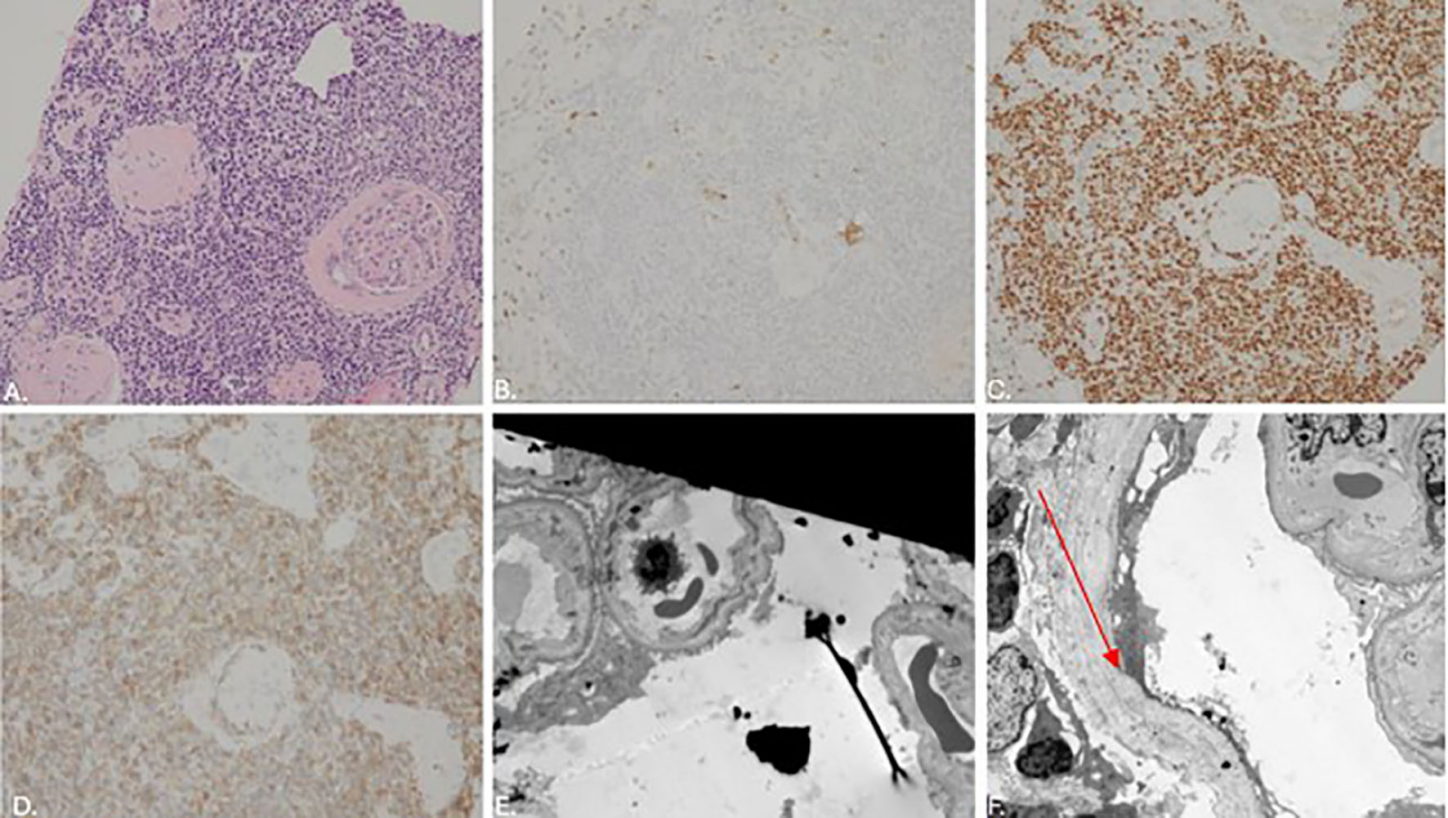
Kidney allograft biopsies in Spring 2025 are shown after staining with Hematoxylin & Eosin (HE, 40x), and CCND1 and CD5 immunohistochemistry (IHC) staining (200x). Immunohistochemical staining shows the majority of interstitial lymphocytes to be neoplastic CD20-positive, PAX-5-positive B-cells with aberrant CD5 expression and kappa restriction; the neoplastic cells are negative for lambda and for cyclin D1. Immunohistochemical staining for CD3 shows admixed, non-neoplastic T-cells. **(A)** Core kidney biopsy with HE staining demonstrating interstitial lymphocytes. **(B)** Interstial lymphocytes are negative for CCND1 **(C)** Interstial lymphocytes are positive for PAX5 **(D)** Interstial lymphocytes are positive for CD5 **(E, F)** EM showing mild effacement of visceral epithelial foot processes, segmental widening of the lamina rara interna (arrow),segmental mesangial interposition, unremarkable endothelium, and a mild increase in mesangial matrix. Findings consistent with transplant glomerulopathy/acute cellular rejection.

Clinically, the patient continued to experience episodes of renal impairment (sCr 2.52 mg/dL), leading to hospital admission for a renal biopsy in spring 2025. The renal biopsy revealed acute antibody-mediated rejection as seen on electron microscopy in **Figures 1E, F**.

**Figure 2:**
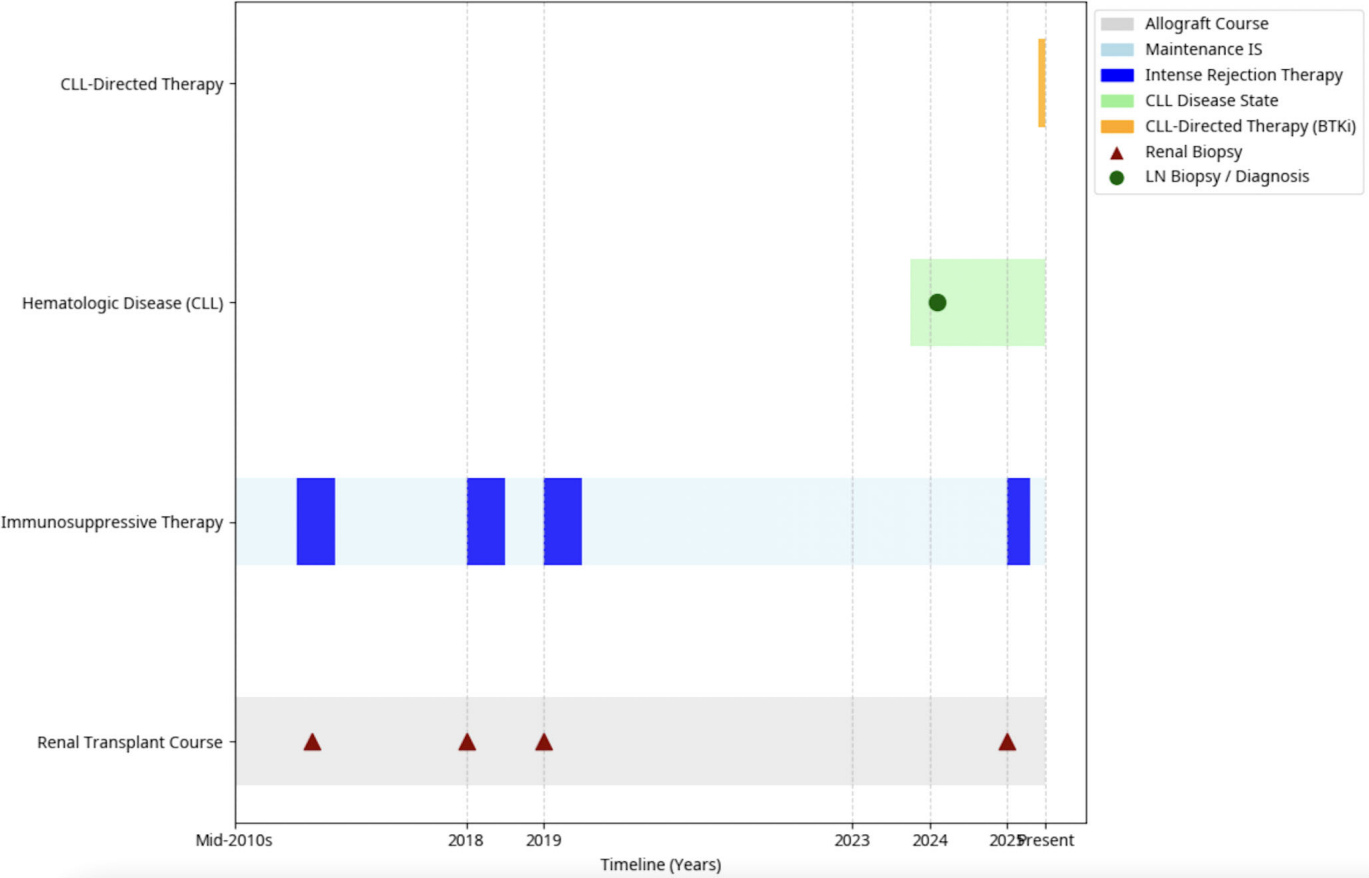
Swimmer plot of renal transplant course and CLL progression.

Immunohistochemical staining showed the majority of interstitial lymphocytes (**Figure 1A**) to be neoplastic CD20-positive, PAX-5-positive B-cells (**Figure 1C**) with aberrant CD5 expression (**Figure 1D**) and kappa restriction; these neoplastic cells were negative for lambda and cyclin D1 (**Figure 1B**). Immunohistochemical staining for CD3 showed admixed, non-neoplastic T-cells. *In situ* hybridization for Epstein-Barr virus was negative. The immunophenotype of the neoplastic cells matched that of the previous right axillary lymph node specimen, thereby confirming renal involvement by CLL in a transplanted kidney. The patient was treated for acute rejection with three doses of methylprednisolone sodium succinate 500mg for 3 doses, IVIG 0.5mg/kg for three doses, and rituximab 375mg/m^2^ for two doses over 14 days. Given evidence of CLL graft infiltration in an extranodal site, treatment with an oral Bruton’s tyrosine kinase inhibitor (BTKi), Zanubrutinib (160 mg twice daily), was initiated in early summer 2025 (**Figure 2**). Two weeks after initiation with BTKi, sCr stabilized at 1.70 mg/dL. The patient did not experience any complications related to Zanubrutinib at her four week follow up with hematology.

## Discussion

Chronic lymphocytic leukemia accounts for almost 25% of all leukemia diagnoses in the United States (1). It is a lymphoproliferative disorder characterized by the build-up of monoclonal mature B lymphocytes (2). According to the latest guidelines from the International Workshop on CLL, the diagnosis of CLL requires either a sustained increase in peripheral blood clonal B lymphocytes exceeding 5,000 cells/mm³ for at least three months, showing characteristic morphology and immunophenotype, or the presence of cytopenias due to clonal B-cell infiltration of the bone marrow regardless if the peripheral B-cell count is below 5,000 cells/mm³ (7).

The Rai and Binet staging systems are the most utilized methods for classifying CLL severity, based on the extent of lymphadenopathy, splenomegaly, hepatomegaly, and peripheral cytopenias (8, 9). Our patient was initially diagnosed with RAI stage 1 CLL. CLL course remains heterogeneous, with approximately 30% of patients exhibiting indolent disease and others progressing to multi-organ involvement (2). Renal involvement of CLL is relatively common and typically not associated with severe clinical outcomes. However, the mechanisms driving this remain unclear. In a study by Barcos et al., autopsies of 109 CLL patients revealed that, following the liver, spleen, and lymph nodes, the kidneys were the most frequently infiltrated organ, observed in 63% of cases (3). In a case series of patients with CLL and kidney involvement, the most common indications for a renal biopsy were acute kidney injury (AKI) and nephrotic syndrome (4). The study found that over 70% of the kidney-related pathological findings were directly attributable to CLL. The most common lesions included membranoproliferative glomerulonephritis, followed by direct CLL infiltration, minimal change disease, and acute interstitial nephritis.

Among six patients where CLL infiltration was the primary cause of renal injury, all presented with moderate to severe renal dysfunction at the time of biopsy (serum creatinine ranging from 149 to 750 μmol/L), despite having fewer than 5,000 leukocytes/mm³ in peripheral blood. Notably, the degree of renal impairment did not correlate with the extent of CLL infiltration. Furthermore, serum creatinine levels improved in all patients who responded to treatment with agents such as rituximab, cyclophosphamide, vincristine, and/or prednisone. Our patient’s renal biopsy showed interstitial neoplastic lymphocytes positive for CD20, PAX-5, CD5, and kappa restriction. Although native kidney involvement in CLL is relatively common, reports of CLL infiltration in kidney allografts remain exceedingly rare (6). A four-patient case series investigated outcomes of patients with CLL after undergoing renal transplant (5). All four patients were diagnosed with CLL before renal transplantation. They were all initially diagnosed with Binet stage A disease. Three out of the four patients experienced progression of CLL. Two out of the four experienced worsening renal function. Renal biopsies showed massive and specific infiltration by CLL in the renal interstitium. One patient lost his graft 14 months after transplant. One patient developed a recurrence of glomerulonephritis.

Most importantly, all patients experienced severe infectious complications. The increased susceptibility to infections is ascribed to the combined effects of post-transplant immunosuppressive therapy and the intrinsic immune dysfunction associated with CLL, including cytopenias and hypogammaglobulinemia. D’ Ythurbide et al. suggests that the presence of CLL should be a critical factor in evaluating transplant eligibility, potentially necessitating adjustments in immunosuppressive therapy and close monitoring post-transplant. Moreover, another study highlighted poor outcomes, primarily due to infectious complications in CLL patients who underwent kidney transplant (4). However, it is essential to note that these cases occurred before the advent of Bruton tyrosine kinase inhibitor (BTKi). The outcomes of CLL patients with renal involvement in the BTK era are unknown. Stavart et al. published a case of clinically significant CLL with biopsy-proven renal involvement treated with BTKi (6). This case underscores the potential of BTK inhibitors in treating CLL-related kidney issues.

Similar to our case, this patient was initially diagnosed with early-stage RAI disease, which did not require treatment. However, renal function deteriorated, prompting another biopsy, which showed focal interstitial infiltration (10–20% total surface) by CLL-like B lymphocytes (PAX5+, CD5+, CD20+, CD23+, surface IgM-; cyclin D1 negative). This patient was started on Ibrutinib, a type of targeted therapy that blocks a protein needed by cancer cells to multiply, which led to stabilization of renal function. A repeat renal biopsy showed the absence of CLL-like B lymphocyte infiltration. Our patient was treated with Zanibrutinib, another BTK inhibitor. To our knowledge, this is the second CLL case involving the kidney treated with BTKi.

## Conclusion

Our case highlights a rare and clinically significant manifestation of CLL involving a renal allograft in a transplant recipient. Despite an indolent early-stage presentation, the disease progressed to extranodal infiltration that impaired graft function. Initiation of zanubrutinib led to clinical stabilization, marking only the second published case of successful BTKi treatment for CLL-infiltrated allografts. Given the immunocompromised status of transplant recipients and the increasing availability of targeted therapies, early recognition of CLL-related renal complications—particularly in the context of allograft dysfunction—is essential. This case underscores the evolving role of BTK inhibitors in managing extranodal CLL and calls for heightened vigilance in transplant populations where overlapping immune risks may mask leukemic progression.

### Patient perspective

“When I was diagnosed with cancer after already getting a transplant, I was concerned how treatment would affect my kidney and if my donor was in some way responsible for me getting cancer now. I was also concerned if I would be needing radiation therapy initially. I felt a sense of relief when I was told that I would not be needing radiation and that my donor was not responsible for me getting cancer now. I felt supported by my oncology and felt like they had my health as their top priority. I felt like I was going with the flow because I felt like the team was doing the right thing. I was very curious why my case was so interesting, and I am glad the team explained it to me. I am hopeful that my case can help other people”.

## Data Availability

The original contributions presented in the study are included in the article/supplementary material. Further inquiries can be directed to the corresponding author.
