# Correction: Case Report: renal allograft infiltration by chronic lymphocytic leukemia successfully treated with zanubrutinib

**DOI:** 10.3389/fonc.2026.1810204

**Published:** 2026-03-02

**Authors:** Brian Abboud, Nehemias Guevara Rodriguez, Arslan Babar, Noemy Coreas, Katherine Robbins, Ranju Kunwor

**Affiliations:** 1 Department of Internal Medicine, SSM Health-Saint Louis University School of Medicine, Saint Louis, MO, United States; 2 Division of Hematology, Oncology and Bone Marrow Transplant, Department of Internal Medicine, SSM-Health Saint Louis University School of Medicine, St. Louis, MO, United States; 3 Department of Gynecologic Oncology, Salvadoran Social Security Institute (ISSS), University of El Salvador (UES), San Salvador, El Salvador; 4 Department of Pathology, SSM-Health Saint Louis University School of Medicine, Saint Louis, MO, United States

**Keywords:** bruton tyrosine kinase (BTK) inhibitor, CLL (chronic lymphocytic leukemia), hematology, immunosuppression, malignant hematologic disease, renal transplant, zanubrutinib, zanubrutinib acute kidney injury as initial manifestation of chronic lymphocytic leukemia/small lymphocytic lymphoma

Author “Noemy Coreas” was erroneously spelled as “Noemy Coery”.

The original version of this article has been updated.

